# A single site for N-linked glycosylation in the envelope glycoprotein of feline immunodeficiency virus modulates the virus-receptor interaction

**DOI:** 10.1186/1742-4690-5-77

**Published:** 2008-08-22

**Authors:** Brian J Willett, Elizabeth L McMonagle, Nicola Logan, Ayman Samman, Margaret J Hosie

**Affiliations:** 1 Retrovirus Research Laboratory, Institute of Comparative Medicine, Faculty of Veterinary Medicine, University of Glasgow, Bearsden Road, Glasgow, G61 1QH, UK

## Abstract

Feline immunodeficiency virus (FIV) targets helper T cells by attachment of the envelope glycoprotein (Env) to CD134, a subsequent interaction with CXCR4 then facilitating the process of viral entry. As the CXCR4 binding site is not exposed until CD134-binding has occurred then the virus is protected from neutralising antibodies targeting the CXCR4-binding site on Env. Prototypic FIV vaccines based on the FL4 strain of FIV contain a cell culture-adapted strain of FIV Petaluma, a CD134-independent strain of FIV that interacts directly with CXCR4. In addition to a characteristic increase in charge in the V3 loop homologue of FIV_FL4_, we identified two mutations in potential sites for N-linked glycosylation in the region of FIV Env analogous to the V1–V2 region of HIV and SIV Env, T271I and N342Y. When these mutations were introduced into the primary GL8 and CPG41 strains of FIV, the T271I mutation was found to alter the nature of the virus-CD134 interaction; primary viruses carrying the T271I mutation no longer required determinants in cysteine-rich domain (CRD) 2 of CD134 for viral entry. The T271I mutation did not confer CD134-independent infection upon GL8 or CPG41, nor did it increase the affinity of the CXCR4 interaction, suggesting that the principal effect was targeted at reducing the complexity of the Env-CD134 interaction.

## Background

The initial event in the process of viral entry is the interaction between the virus and its cellular receptor. For HIV-1, the trimeric Env complex comprising gp120 and gp41 attaches to the primary viral receptor CD4 [1,2] on the surface of the target cell. This interaction is believed to induce a conformational change in gp120 that leads to exposure of the binding site for the coreceptor, usually the chemokine receptors CXCR4 and CCR5 [3,4]. Engagement of the coreceptor triggers a further conformational change in the Env complex that results in exposure of the gp41 fusion domain and initiates the process of fusion of the viral and cellular membranes. Given that the virus-receptor interaction initiates the process of viral entry, the binding sites on gp120 for the primary and co-receptors should, logically, make good targets for neutralising antibodies. Indeed, the monoclonal antibody (MAb) b12 [5] targets the CD4 binding site on gp120 and has broad neutralising activity against diverse isolates of HIV-1 while MAbs such as 17b, target the chemokine receptor binding site, engaging the Env complex post-attachment to CD4 (a "CD4-induced" epitope). During natural infection antibodies targeting the CD4 binding site are seldom elicited [6]; the CD4 binding site is recessed in gp120, partially occluded by the hypervariable loops and protected by "conformational masking" [7-9]. When such antibodies are elicited, they display potent, broad neutralizing activity [6]. In contrast, although the co-receptor binding site is not exposed on the virion until after CD4 binding has occurred, antibodies targeting the co-receptor binding site are common in sera from HIV-infected patients, and in the presence of soluble CD4 display potent cross-clade neutralising activity [10].

Some strains of HIV and SIV are capable of by-passing the primary receptor and interacting directly with the co-receptor [11-15]. In these "CD4-independent" strains of virus, the chemokine receptor binding site may be more exposed [16] and as such, they may be more sensitive to neutralising antibodies than their CD4-dependent counterparts [11,16-19]. Accordingly, the humoral immune response may exert a strong selective pressure against the emergence of CD4-independence *in vivo*: CD4-independent strains would have a broader cell tropism *in vivo*, assisting with viral dissemination into cellular compartments where CD4 expression may be low, for example the CNS [20-22]. The inextricable link between receptor usage, cell tropism and neutralisation sensitivity [23,24] may advise the design of novel immunogens for HIV vaccination. Deletion of the V2 hypervariable loop from HIV-1 SF162 renders the virus susceptible to virus neutralisation [25]. Antibodies raised against the SF162ΔV2 immunogen target the CD4-binding site preferentially suggesting that the V2 loop deletion exposes the CD4-binding site. The V2-loop deletion in SF162ΔV2 eliminates a site for N-linked glycosylation and studies with HIV-1 ADA have shown that loss of a single N-linked glycan from HIV-1 ADA gp120 switches the virus from a CD4-dependent to CD4-independent phenotype [12] by re-positioning the V1/V2 loops. Similarly, mutation of glycosylation sites in SIVmac239 Env enhance CD4-independent infection mediated by CCR5 [26]. Taken together, these data indicate it may be possible to manipulate the humoral immune response towards the CD4 binding site by modulating the surrounding environment on gp120 and in doing so, create an immunogen that will induce broadly neutralising antibodies.

Feline immunodeficiency virus (FIV) is a widespread pathogen of the domestic cat and in its natural host species it induces a disease state similar to AIDS in human beings. FIV targets CD4+ helper T cells by binding to CD134 (also known as OX40) [27], a member of the tumour necrosis factor receptor superfamily that is expressed selectively upon feline helper T cells [28,29]. All primary strains of FIV tested to date, utilise CD134 as a receptor for viral entry [27,29,30], however cell culture-adapted strains of FIV such as Petaluma F14 and 34TF10 [31,32] are able to infect and form syncytia in cell lines in the absence of CD134 [27]. CD134-independent FIV infection is mediated by a direct interaction with CXCR4 [33,34], analogous to infection with CD4-independent strains of HIV [13]. However, over-expression of CXCR4 alone is sufficient to render cells susceptible to infection with some strains of FIV [35], suggesting that such strains of virus may have a propensity to adaptation in cell culture to CD134-independence.

Whole inactivated virus vaccines derived from the FL4 cell line (a cell line infected persistently with the Petaluma strain of FIV [36]) induce both humoral and cellular immunity and offer a degree of protection against challenge with heterologous strains of virus [37-42]. Accordingly, the FL4 cell line has provided the basis for the first commercially available FIV vaccine (Fel-O-Vax FIV, Fort Dodge), approved for use in the USA, Japan, New Zealand and Australia. The vaccine has attracted a degree of controversy as independent experiments have failed to demonstrate protective efficacy against heterologous challenge [43], addressing these conflicting reports is of importance to advancing lentiviral vaccine development [44]. Previously, Env-based immunogens derived from primary strains of FIV failed to induce protective immunity, and in some cases led to an accelerated viraemia following challenge [45-51]. In order to inform the design of lentiviral vaccines that will induce broadly neutralising antibodies, we examined the FL4 virus for the presence of novel features acquired during the process of adaptation to cell culture that may have contributed to its enhanced immunogenicity. Here, we identify a potential site for N-linked glycosylation that is highly conserved among field isolates of FIV but which is absent from the FL4 Env. By introducing similar mutations into primary isolates of FIV, we demonstrate that N-linked glycosylation at this site impacts on the virus-receptor interaction. These findings bear striking similarities to observations with both HIV and SIV [52-57] and may provide an insight into the mechanism by which the FL4 immunogen induces virus neutralising antibodies.

## Methods

### DNA constructs and mutagenesis

The FL4 strain of FIV lacks sites for N-linked glycosylation at Asn-269 due to a threonine to isoleucine switch at 271 (T271I), and Asn-342 due to an asparagine to tyrosine substitution at 342 (N342Y). We mutated these sites in the GL8 and CPG41 Envs by amplification using a 5' primer from the leader-SU junction 5-TAGACGCGTAAGATTTTTAAGGTATTC (5' MLU) and either 5'-CGAGATATTATAACAGATGTTATTAGCACAT-3' (ENV 7076) or 5' GGTCTTGAATCTGTGAAGTGTACCACATA (ENV 7288). The amplification products were purified by agarose gel electrophoresis (QIAEx gel extraction kit, QIAGen, UK) and used as 5' primer in conjunction with a 3' primer from the RRE region 5'-AATGGATTCATATGACACATCTTCCTCAAAGGG (3' NDE) to amplify the full-length SU-TM products. The mutated Envs were sub-cloned into the GL8_MYA _molecular clone as Mlu-I/Nde-I fragments as previous [58]. The double mutant (Asn-269 and Asn-342) was generated by amplification firstly using 5'MLU and ENV-7076, extending using ENV-7288 and then finally amplifying the entire Env using the double-mutated fragment and 3'NDE primer. The sequences of each construct were confirmed using a BigDye^® ^Terminator v1.1 cycle sequencing kit (Applied Biosystems) followed by analysis on an Applied Biosystems 3700 genetic analyser.

### Cells and viruses

MYA-1 [59] cells and MCC [60]-derived cell lines were cultured in RPMI 1640 medium. 293T were maintained in Dulbeccos modification of Eagle's medium (DMEM). All media were supplemented with 10% foetal bovine serum (FBS), 2 mM glutamine, 0.11 mg/ml sodium pyruvate 100 IU/ml penicillin, 100 μg/ml streptomycin. The medium for MYA-1 cells was supplemented with conditioned medium from a murine cell line (L2.3) transfected with a human IL-2 expression construct (kind gift of M. Hattori, University of Tokyo) at a final concentration equivalent to 100 U/ml of recombinant human interleukin-2 (IL-2), and 50 μM 2-mercaptoethanol. All media and supplements were obtained from InVitrogen Life Technologies Ltd. (Paisley, UK). Cell lines expressing CD134 and the chimaeric constructs were maintained in G418 (InVitrogen, Paisley, UK).

Molecular clones carrying the Asn-269 (T271I), Asn-342 (N342Y) or the Asn-269/Asn-342 double mutant (Δ2N) were transfected into 293T cells using Superfect (QIAGen) and recovered by co-culture with MYA-1 cells at 72 hrs post-transfection. Viruses were expanded in MYA-1 cells before harvesting, 0.45 μm filtration and storage at -80°C.

### Polyacrylamide gel electrophoresis (PAGE) analyses

1.0 to 1.5 × 10^7 ^infected MYA-1 cells were washed by centrifugation (1000 rpm, 5 mins.) through ice-cold phosphate buffered saline and resuspended in lysis buffer comprising 1% CHAPS in 10 mM Tris (ph 7.4), 150 mM sodium chloride, 2 mM ethylenediamine tetraacetic acid and supplemented with a Complete™ protease inhibitor tablet (Roche Applied Science, Burgess Hill, UK). Lysates were mixed with reducing Laemmli sample buffer [61] and separated on either 4–15% polyacrylamide gels (Ready-Gel, Biorad, Hemel Hempstead, UK) or 10% polyacrylamide gels (prepared as previously [62]). Separated proteins were transferred to nitrocellulose by electroblotting (iBlot™, Invitrogen); viral antigens were detected using pooled polyclonal cat serum from FIV infected cats followed by biotinylated goat anti-cat IgG, or monoclonal antibody vpg71.2 followed by biotinylated goat anti-mouse IgG conjugates (Vector Laboratories Ltd., Peterborough, UK). Bound conjugate was revealed using the Vectastain ABC kit and 5-bromo-4-chloro-3-indolyl phosphate/nitroblue tetrazolium substrate (Vector Laboratories Ltd.).

### Preparation of HIV (FIV) pseudotypes

FIV *env *gene expression constructs GL8, B2542, CPG41 and PPR have been described previously [27,30,63]. 5 μg of each VR1012-*env *and 7.5 μg of pNL4-3-Luc-E^-^R^- ^were co-transfected into HEK-293T cells using SuperFect activated dendrimer (QIAgen) as per manufacturer's instructions. The nomenclature "HIV(FIV)" denotes an FIV Env protein on an HIV particle. Culture supernatants were collected at 48 hours post-transfection, filtered at 0.45 μm and frozen at -80°C until required. Target cell lines were seeded at 5 × 10^4 ^cells per well of a CulturPlate™-96 assay plate (Perkin Elmer, Life and Analytical Sciences, Beaconsfield, UK) and used immediately. The cells were then infected with 50 μl of HIV (FIV) luciferase pseudotypes, cultured for 72 hours and then luciferase activity quantified by the addition of 50 μl of Steadylite HTS™ (Perkin Elmer) luciferase substrate prior to measurement by single photon counting on a MicroBeta TriLux luminometer (Perkin Elmer).

### Virus neutralisation assays

Sera were diluted 5-fold in MYA-1 culture medium and then 25 μl of each dilution (in triplicate) was incubated with 25 μl of HIV(FIV) luciferase pseudotype, incubated for one hour at 37°C and then added to 50 μl (5 × 10^4 ^cells) of CLL-CD134 cells per well of a CulturPlate™-96 assay plate (Perkin Elmer, Life and Analytical Sciences, Beaconsfield, UK). The cells were then cultured for 72 hours and luciferase activity quantified by the addition of 100 μl of Steadylite HTS™ (Perkin Elmer) luciferase substrate and measurement by single photon counting on a MicroBeta luminometer (Perkin Elmer).

### Growth of FIV *in vitro*

The growth of FIV *in vitro *was assessed in MYA-1 cells. Supernatants were collected every three days and assayed for reverse transcriptase (RT) activity using Lenti-RT non-isotopic RT assay kit (Cavidi Tech., Uppsala, Sweden). RT values were then calculated relative to purified HIV-1 RT standard.

### Syncytium formation in adherent cells

AH927 cells stably transduced with pDONAI vector only, or with vector encoding fCD134, fCRD1xhCD134 or hCD134, were transfected with each *env *construct using Superfect (Qiagen, Crawley, UK) and incubated for 48 hours at 37°C. The cells were then fixed and stained with 1% methylene blue/0.2% basic fuchsin in methanol and photographed using a Leica DMLB microscope (Leica Microsystems (UK) Ltd., Milton Keynes) and Photometrics SenSys digital camera (Photometrics Ltd., Tucson, USA).

### Inhibition of viral entry

1 × 10^5 ^MYA-1 or CLL-CD134 cells were incubated with the CXCR4 antagonist AMD3100 [64-66] in complete medium in 96-well culture-treated luciferase assay plates (CulturPlate™ 96) for 30 minutes at 37°C. Viral pseudotypes were then added and the plate returned to the 37°C incubator. Cultures were maintained for 72 hours post-infection at which point 100 μl of Steadylite HTS™ (Perkin Elmer) luciferase substrate were added and luminescence measured by single photon counting on a MicroBeta luminometer (Perkin Elmer).

### Production of recombinant IgG-Fc fusion proteins

FIV Env SU-Fc fusion proteins were produced by amplifying the SU coding sequence with the oligonucleotide primers 5'-CGATCTAGAAACAATAATTATGGCAGAAG-3' and 5'-GGCGGCCGCTGGTACCAC(C/T)AAGTAATC-3' corresponding to the start codon for Env leader sequence and the SU/TM junction respectively. The amplified products were cloned as XbaI/NotI fragments into pTorsten [67], expressed in CHO cells in CELLine AD1000 bioreactor flasks (Integra Biosciences (Scientific Laboratory Supplies, Nottingham, U.K.)) in medium supplemented with low IgG serum (Integra Biosciences). Culture supernatant was filtered at 0.22 μm and frozen at -80°C prior to use in order to preserve optimal bioactivity. Proteins were purified from culture supernatant as previous [28].

## Results

### Characterisation of FIV FL4 SU

The FL4 cell line is a feline lymphoblastoid cell line that is persistently infected with the Petaluma strain of FIV [68]. The virus derived from FL4 cells provided the substrate for whole inactivated virus vaccines that conferred strain-specific immunity to infection with FIV [37,38,41], and, in combination with the subtype D Shizuoka strain, the basis for Fel-O-Vax FIV (Fort Dodge Animal Health). The predicted amino acid sequence of the FL4 vaccine strain was compared with that of the prototypic F14 and 34TF10 clones of the Petaluma isolate of FIV [31,69] in order to identify non-synonymous mutations in gp120 that may have been acquired during the process of cell culture adaptation. Unique amino acid substitutions in SU were identified at T271I, N342Y, W347R and F388L (amino acid numbering is relative to F14 [31]). Of these substitutions, T271I and N342Y ablated potential sites for N-linked glycosylation in SU. We asked whether the T271I, N342Y substitutions were observed in field isolates by comparing the FL4 sequence with published sequences using the BLAST search program [70]. The potential site for N-linked glycosylation targeted by the N342Y mutation was unique to FL4 (in comparison with 35 published amino acid sequences) while the N-linked glycosylation site targeted by the T271I mutation was absent in only three other sequences (n = 34); Aomori 1 and 2 [71] (two related Japanese isolates) and FC2 [72]. Given that the T271I and N342Y substitutions were uncommon amongst field isolates and present in neither the F14 and 34TF10 molecular clones, nor the parent biological isolate of FIV Petaluma (not shown), we hypothesised that these mutations may have either been acquired during the process of cell culture adaptation, or amplified from an initial quasispecies during cell culture. Similar adaptations have been shown previously to affect receptor usage for lentiviruses and to alter neutralisation sensitivity and immunogenicity [25,52,73].

**Figure 1:**
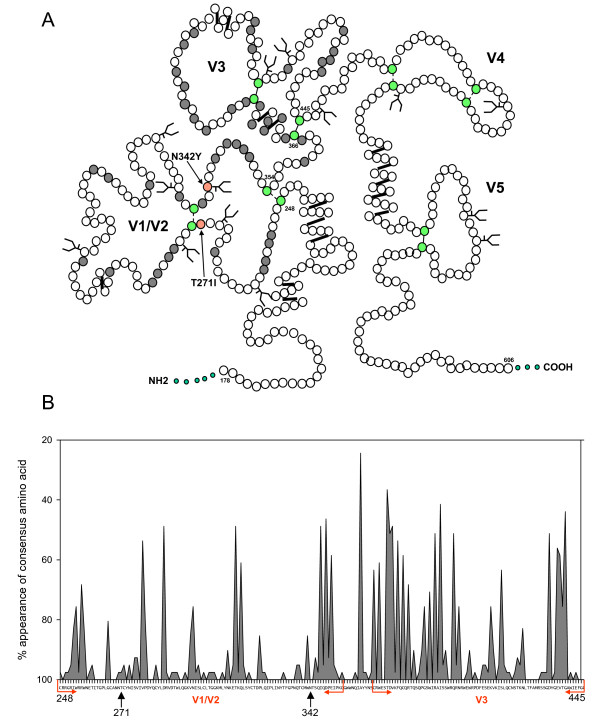
A. Schematic structural model of the FIV SU proteins illustrating the locations of the T271I and N342Y mutations (red), conserved cysteine residues (green) and predicted sites for N-linked glycosylation (). Residues in the V1/V2 or V3 homologues displaying >10% variation from the consensus sequence are shaded. B. Position-specific scoring matrix (PSSM) analysis of amino acids 248 to 445 of FIV SU encompassing the homologous regions to HIV V1/V2 and V3 illustrating the likelihood of the consensus amino acid appearing in an SU sequence. T271 and N342 are arrowed.

We next examined the predicted locations of T271I and N342Y substitutions on the FIV SU protein, comparing the locations with schematic structural models for FIV SU and HIV SU based on predictive algorithms for secondary structure [74], disulphide bond architecture [75,75,76] and informed by the solved crystal structure of HIV Env [7,8]. T271I and N342Y are predicted to lie in a region of FIV SU which may be analogous to the area at the base of the HIV V1 and V2 stem (Fig. 1A). For the purpose of this study, and to assist with direct comparisons between FIV and HIV, this region is referred to as the V1/V2 homologue herein. Although this region of FIV Env was thought formerly to be relatively conserved, a reappraisal based on accumulated Env sequence data indicates pockets of variability within this region, as illustrated when variability is plotted using consensus position-specific scoring matrix analysis [77] (PSSM, Fig. 1B). Mutations in the V1/V2 region of HIV SU affect the interaction between the HIV SU and its receptor and co-receptor(s) and alter the antigenicity of the envelope glycoprotein, promoting the production of antibodies targeting the CD4 binding site [12,73,78,79]. Similarly, for HIV loss of N-linked glycans from this region contribute to CD4-independence [12].

### Incorporation of the T271I and N342Y mutations into primary isolates of FIV

**Figure 2:**
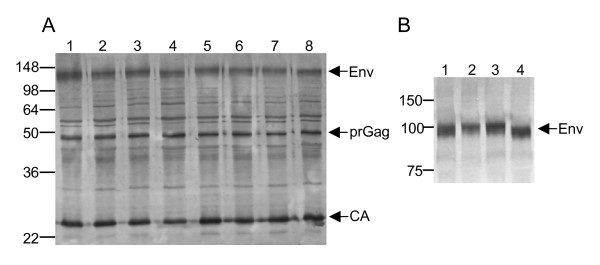
Mutant proviral clones bearing T271I or N342Y yield infectious virus. A. Pelleted virus from MYA-1 cells infected with GL8 (1–4) and CPG41 (5–8) viruses bearing WT (1,5), T271I (2,6), N342Y (3,7) or Δ2N (4,8) were separated by reducing SDS-PAGE on a 12% acrylamide gel, transferred to nitrocellulose and probed with pooled cat anti-FIV. Approx. molecular mass (kDa) are on left, primary reactivities Env, prGag and CA arrowed on right. B. 10–20% gradient gel analysis of GL8 WT (1), T271 (2), N342Y (3) and Δ2N (4) probed with anti-FIV (subtype-specific) SU monoclonal antibody vpg71.2. Approx. molecular mass (kDa) on left, Env is arrowed on right.

The T271I and N342Y mutations were reproduced in molecular clones of FIV GL8 and a chimeric molecular clone bearing the CPG41 Env in the GL8 backbone by site-directed mutagenesis. Constructs were transfected into 293T cells and replication competent virus was recovered into IL2-dependent feline T cells (MYA-1). All viruses replicated with similar efficiency in MYA-1 cells (not shown). Bulk supernatants were prepared, virus pelleted by ultracentrifugation and analysed by SDS-PAGE. Immunoblotting using polyclonal anti-FIV (Fig. 2A) confirmed that similar levels of viral proteins were produced by each virus. Although a marginal reduction in the apparent size of Env was suggested in the Δ2N mutants of both GL8 and CPG41 (Fig. 2A), this was visualised more readily when the GL8 viruses were separated on a 10–20% gradient gel (Fig. 2B) and probed with the subtype-specific monoclonal antibody vpg71.2 (vpg71.2 does not recognise CPG41 Env). Under these conditions, a clear downward shift could be discerned in the Δ2N double mutant virus but in neither of the single mutants.

### Effect of the T271I and N342Y mutations on receptor usage by GL8 and CPG41

**Figure 3:**
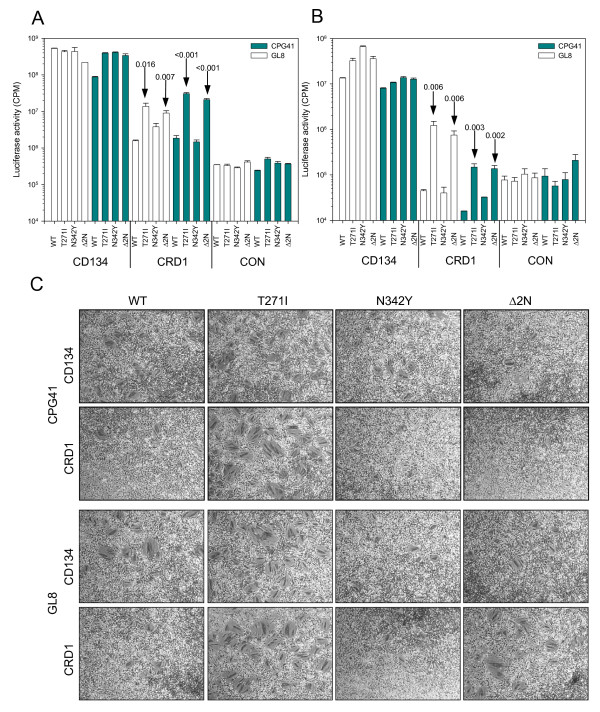
Infection of the feline cell lines AH927-FX4P [81] (A) and MCC [27] (B) stably-transduced with retroviral vectors encoding full-length fCD134 (CD134), CRD1 of fCD134 in the context of hCD134 (CRD1) or vector only control (CON). Infection of both cell lines expressing CRD1 was enhanced significantly in the T271I and Δ2N Envs. Histograms display the mean +/-SE (n = 3), arrows show P-values, t-test. C.) Syncytium formation in AH927-FX4P cells expressing feline CD134 (CD134), or feline CD134 CRD1/human CD134 chimera (CRD1). Cells were transfected with eukaryotic expression vectors bearing the GL8 or CPG41 *envs *carrying the WT, T271I, N342Y or Δ2N sequences. Cells were fixed and stained at 48 hours post-transfection, representative fields are shown.

FIV GL8 and CPG41 require determinants in both CRDs 1 and 2 of CD134 for viral entry while infection with strains such as PPR and B2542 is mediated by CRD1 alone [30,63]. Accordingly, a chimeric CD134 molecule comprising CRD1 of feline CRD1 in the context of human CD134 may be used to differentiate strains such as GL8 and CPG41 from strains such as PPR and B2542 in viral entry assays (reviewed in [80]). The feline cell lines AH927-FX4P [81] (Fig. 3A) and MCC [27] (Fig. 3B) stably expressing full-length feline CD134 (CD134) or a chimeric receptor bearing feline CD134 CRD1 in the context of human CD134 (CRD1), or transduced with vector only (CON) were infected with HIV (FIV) luciferase pseudotypes bearing the wild type (WT), T271I, N342Y or Δ2N Envs derived from GL8 and CPG41 and viral entry was assessed. Pseudotypes bearing either the WT or mutant Envs infected AH927-FX4P (Fig. 3A.) and MCC (Fig. 3B.) cells expressing CD134 with comparable efficiency. Infection of CRD1-expressing cells was diminished greatly for both GL8 and CPG41 WT Envs, consistent with previous data [30,63]. The N342Y mutants behaved ostensibly the same as the WT Envs, demonstrating a marked preference for CD134 over the CRD1 chimera. In contrast, introduction of the T271I and Δ2N Envs partially restored the ability of the viruses to use the CRD1 chimera (significant enhancement, t-test). The effect was most stark on MCC cells (Fig. 3B.) where GL8 WT infection of MCC-CRD1 was 300-fold less efficient, whereas infection with the T271I and Δ2N mutants was reduced by only 27 and 47-fold respectively, suggesting that the presence of the T271I mutation, but not the N342Y mutation, facilitated the interaction between the GL8 Env and CRD1 of CD134.

If the T271I mutation conferred enhanced usage of the CRD1 chimera as a receptor then we would predict that direct transfection of the T271I mutant into AH927-FX4P cells expressing the CRD1 chimera would result in enhanced syncytium formation compared to the wild type. We therefore compared the ability of the GL8 and CPG41 WT, T271I, N342Y and Δ2N Envs to induce syncytium formation in AH927 cells expressing CD134, the CRD1 chimera or vector only. Introduction of the T271I mutation enhanced syncytia formation in both CD134 and CRD1 chimera expressing cells (Fig. 3C). Few syncytia were observed in the CRD1 chimera-expressing cells transfected with GL8 or CPG41 WT Envs. The N342Y Envs induced fewer, and smaller syncytia in CD134-expressing cells than the WT Envs and few syncytia in the CRD1-expressing cells. Surprisingly, while HIV (FIV) pseudotypes bearing the GL8 and CPG41 Δ2N Envs behaved essentially the same as the pseudotypes bearing the T271I mutant Envs in the entry assay (Fig. 3A and 3B), in the syncytial assay, the Δ2N mutant Envs showed a marked reduction in syncytia formation compared with the T271I mutants. Similar results were observed with both the GL8 and CPG41 series of Envs indicating that in the presence of the N342Y mutation the enhancement of syncytium formation conferred by T271I was lessened. Neither the wild type nor the mutant Envs induced syncytium formation in the vector-only control cells (not shown). The data suggest that the potential N-linked glycosylation sites at positions 269 (NNT) and 342 (NTS) impact on the interaction between the viral Env and its receptor(s).

### Effect of glycosylation site ablation on sensitivity to CXCR4 antagonist

**Figure 4:**
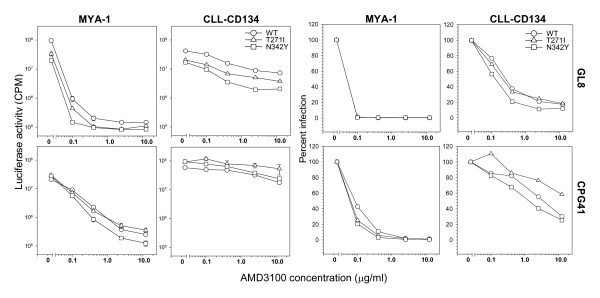
Sensitivity of glycosylation mutants to CXCR4 antagonist. Infection of MYA-1 and CLL-CD134 cells with HIV (FIV) pseudotypes bearing WT, T271I or N342Y mutant GL8 and CPG41 Envs was quantified in the presence of increasing concentrations of CXCR4 antagonist AMD3100. Infection is expressed as luciferase activity (CPM) and percent infection relative to control (no antagonist). Each point represents the mean +/- SE of triplicate samples.

The enhanced usage of the CD134-CRD1 chimaera by the T271 mutants may have arisen due to a less stringent interaction between FIV Env and CD134, the N-linked glycosylation site at 269 contributing to the receptor binding surface. Alternatively, removal of the N-linked glycosylation may have enhanced the ability of the Env to interact with CXCR4, either facilitating partial CD134-independent entry or by enhancing the affinity for CXCR4. To address the latter possibility, we examined the sensitivity of viral pseudotypes bearing the WT, T271I or N342Y Envs to the CXCR4 antagonist AMD3100 (Fig. 4). Sensitivity to AMD3100 was assessed on MYA-1 T cells (expressing low levels of CXCR4 [82]) and CLL-CD134 (expressing high levels of CXCR4 (Willett et al., unpublished data). Where CXCR4 was limiting (MYA-1 cells), infection with both GL8 and CPG41 was inhibited efficiently by AMD3100. Neither the T271 nor the N342Y mutation enhanced or reduced sensitivity to AMD3100 although it was notable that GL8 was significantly more sensitive to antagonism by AMD3100 than CPG41 (at 0.1 μg/ml AMD3100 there was a 100-fold reduction in GL8 entry compared with 5-fold for CPG41), suggesting that CPG41 has a higher affinity for CXCR4 *per se*. Where CXCR4 was abundant (CLL-CD134), inhibition of infection required significantly higher concentrations of AMD3100 and even at 10 μg/ml antagonist, the degree of inhibition never exceeded 80%. The data suggest that the T271I and N342Y mutations do not alter the affinity of the CXCR4 interaction significantly.

### T271I does not induce CD134-independence

**Figure 5:**
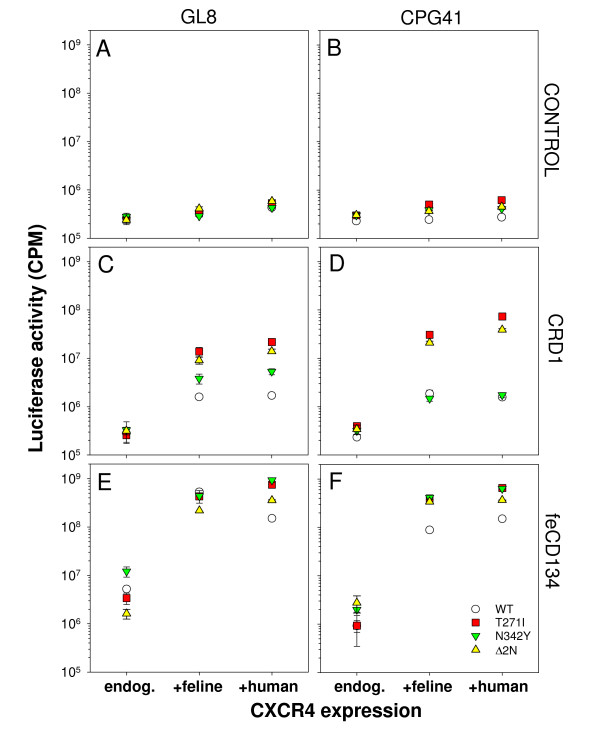
Effect of ectopic CXCR4 expression on infection with glycosylation mutants. AH927 cells expressing low level endogenous CXCR4 (endog.) or enhanced levels of either feline CXCR4 (+feline) or human CXCR4 (+human), in conjunction with feline CD134 (feCD134), CRD1 of fCD134 in the context of hCD134 (CRD1), or vector-only control (Control), were infected with HIV(FIV) pseudotypes bearing the GL8 or CPG41 Envs. 72 hours post-infection luciferase activity was assayed. Each point represents the mean +/- SE of triplicate samples.

We next asked whether the T271I or N342Y mutations conferred CD134-independence upon the GL8 and CPG41 Envs. Feline AH927 cells stably transduced with vectors encoding feline CD134 or the CRD1 chimaera, or the empty expression vector (pDONAI) only, were transduced for a second time with vectors encoding feline CXCR4 or human CXCR4, or expression vector (pBabePuro only). Stable lines were selected that expressed either low level endogenous feline CXCR4 (Fig. 5, endog.)), high level feline CXCR4 (+feline), or high level human CXCR4 (+human) in conjunction with either the CRD1 chimaera or native CD134. The cells were then infected with HIV(FIV) luciferase pseudotypes bearing the GL8 and CPG41 Envs (wild type (WT), T271I, N342Y or Δ2N double mutant) and infectivity assayed (Fig. 5). If the T271I or N342Y mutations conferred CD134-independence, enhanced CXCR4 expression alone would be sufficient to promote viral entry. Control cells expressing endogenous CXCR4 were refractory to infection with all viruses (Fig. 5A,B, "endog.") and ectopic expression of CRD1 did not enhance infection (Fig. 5C,D, "endog."). In contrast, ectopic expression of feCD134 (Fig. 5E,F, "endog.") enhanced infection of the control with all viruses (>10-fold) suggesting that sufficient endogenous CXCR4 is present to facilitate viral entry in the presence of native primary receptor. The addition of exogenous feline or human CXCR4 alone did not enhance infection of the control cells mediated by either the GL8 or CPG41 Envs significantly, irrespective of whether WT, T271I, N342Y or Δ2N (Fig. 5A,B, control). In contrast, the addition of exogenous CXCR4 in the presence of either the feline CD134 CRD1 chimera (Fig. 5C,D, CRD1), or native feline CD134 (Fig. 5E,F, CD134) revealed a significantly more marked enhancement of infection with the T271I and Δ2N viruses than with the WT or N342Y viruses. The data are consistent with the T271I mutation removing the requirement of the viral Env for determinants in CRD2 [30,63], and thus improving its ability to utilise the CRD1 chimaera as an effective viral receptor. Accordingly, the data suggest that the N-linked glycosylation site ablated by the T271I mutation either contributes to the binding site on FIV Env for CD134, or constrains the conformation of Env such that it may only interact with native feline CD134.

### Binding of soluble Fc-SU fusion proteins to CD134-expressing cells

**Figure 6:**
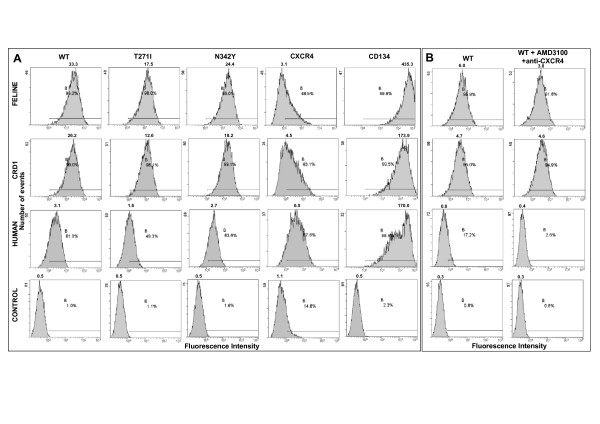
Binding of FIV Fc-SU fusion proteins to CD134-expressing cells. (A) MCC cells stably-transduced with retroviral vectors encoding full-length fCD134 (Feline), CRD1 of fCD134 in the context of hCD134 (CRD1), human CD134 (Human) or vector only control (CON) were incubated with Fc-SU fusion proteins derived from GL8 WT or glycosylation site mutants T271I and N342Y and bound protein detected using PE-anti-human IgG Fc by flow cytometry. CXCR4 and CD134 expression were assayed using anti-CXCR4 (44701) and CD134 (7D6) respectively followed by PE-anti-mouse IgG. (B) Inhibition of residual WT GL8 Fc-SU binding to human CD134 expressing cells by anti-CXCR4. Cells were pre-incubated with AMD3100 and 44701 prior to binding of WT Fc-SU and detection of bound protein by flow cytometry using PE-anti-human IgG Fc. Histograms show 10,000 events and are representative of at least two independent experiments.

Previous studies have demonstrated that soluble IgG-Fc-FIV SU fusion proteins (Fc-SU) recapitulate the receptor binding specificity of the parent virus [29,83-85]. We therefore asked whether GL8 Fc-SU fusion proteins (proteins that exist primarily as dimers) derived from the WT, T271I or N342Y Envs (trimeric on the native virions) would display the receptor binding properties of their respective parent viruses. WT, T271I or N342Y GL8 Env Fc-SUs bound to feline CD134 expressing MCC cells (Fig. 6A, row 1) but not to control MCC cells (Fig. 6A, row 4), consistent with the usage of feline CD134 as a receptor on MCC cells (Fig. 3B). However, a similar level of binding was detected to cells expressing only CRD1 of feline CD134 (Fig. 6A, row 2) suggesting that when the Envs were expressed in soluble form as Fc-SU fusion proteins, the requirement for determinants in CRD2 of CD134 for receptor binding is lost. These data would suggest that the specificity of receptor binding of the GL8 Env may be dependent on intermolecular interactions in the tertiary complex of the native trimeric Env on virions and accordingly that the T271I mutation modulates this interaction. As an additional control for binding specificity, we assessed the binding of Fc-SU proteins to MCC cells expressing human CD134. Neither the WT nor the T271I and N342Y Envs support infection through human CD134 ([30] and data not shown), however, significant (albeit weak) binding of the WT, T271I and N342Y Fc-SU proteins to human CD134-expressing MCC was detected (Fig. 6A, row 3). It was notable that the MCC-derived cell lines transduced with the feline CD134, CRD1 or human CD134 expression vectors showed up-regulated expression of CXCR4 (48.5%, 63.1% and 87.6% respectively) relative to vector-only control cells (14.8%) indicating that the low level binding of the Fc-SU proteins to human CD134 may not have been mediated by human CD134, rather it could have reflected the marked up-regulation of endogenous CXCR4 on these cells. Accordingly, when we repeated the binding of WT Fc-SU to the four cell lines following pre-treatment of the cells with the CXCR4 antagonist AMD3100 and in the presence of anti-CXCR4 monoclonal antibody 44701 (Fig. 6B.), the low level of background binding of Fc-SU to CXCR4 was reduced from 17.2% to 2.6%, consistent with a direct interaction between the soluble SU and CXCR4, as has been noted previously for both FIV and HIV [82,86]. Given that CXCR4 expression alone does not confer susceptibility to infection with WT GL8, and that WT GL8 does not infect cells expressing CRD1 alone, these data suggest that the Fc-SU fusion proteins are in a distinct conformation that fails to mimic that found on native virions.

### Effect of the T271I and N342Y mutations on sensitivity to virus neutralising antibody

**Figure 7:**
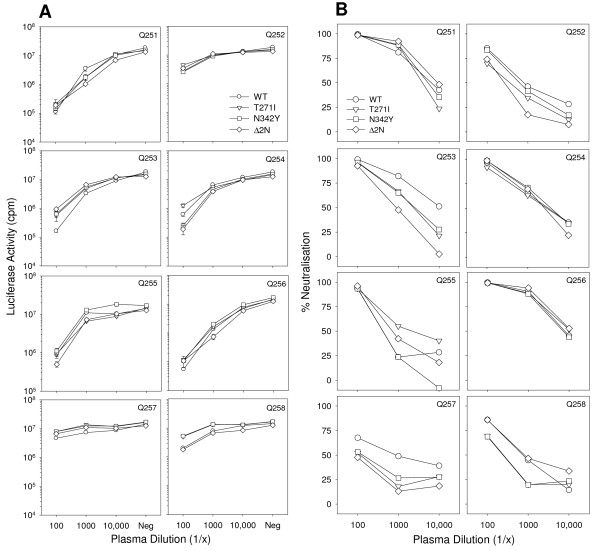
Sensitivity of GL8 WT and T271, N342Y and Δ2N mutants to virus neutralising antibody. HIV (FIV) pseudotypes bearing the WT, T271, N342Y or Δ2N Envs were incubated with 1/100, 1/1000 or 1/10,000 dilutions of plasma from 8 GL8-infected cats (Q251-258), or a no plasma negative control (neg), and plated onto CLL-CD134 cells. Luciferase activity was assayed at 72 hrs post-infection. (A) Luciferase activity (counts per minute), each point represents the mean of three replicates +/- SE, (B) Percent neutralisation relative to no plasma control.

The predicted N-linked glycosylation sites ablated by the T271I and N342Y mutations are highly conserved among field isolates of FIV and reinstated rapidly (as early as 1 month post-infection) following *in vivo *replication of mutant virus [87]. These data suggest that the predicted N-link glycosylation sites at N269 and N342 are critical for maintaining the replicative capacity of the virus *in vivo*. It is possible that glycosylation at these sites protects the virus from virus neutralising antibody (VNA), thus the sensitivity of HIV(FIV) pseudotypes bearing the wild type GL8 Env or the T271I, N342Y and Δ2N mutants to sera from 8 cats infected with GL8 (4 years post-infection) was compared. Of the 8 sera evaluated, 5 sera (Q251, Q253, Q254, Q255 and Q256) contained potent virus neutralising activity (>90% neutralisation at 1/100, Fig. 7) while more modest neutralising activity was detected in sera from Q252, Q257 and Q258 (Fig. 7). There was no consistent difference in sensitivity to VNA between the WT and the T271I, N342Y and Δ2N mutants, suggesting that this region is not a major target for the humoral response to wild type virus.

## Discussion

The FL4 strain of FIV was derived by long-term *in vitro *culture of mixed peripheral blood lymphocytes from cats infected with the Petaluma strain of FIV [68]. The IL-2-independent cell line (FL4) derived from this mixed cell culture stably produced FIV particles at a high level that, following concentration and paraformaldehyde inactivation, provided the first effective vaccine against FIV infection [37], and the basis for the first commercial FIV vaccine, Fel-O-Vax FIV. The FL4 Env would appear to be both stable in its attachment to the viral particle during purification, and highly immunogenic. Here we show that the FL4 virus lacks predicted sites for N-linked glycosylation that are highly conserved in the majority of field strains of FIV. The location of these mutations, T271I and N342Y, is strikingly similar to previously described N-linked glycosylation sites in the Env of SIV and HIV that may contribute to enhanced immunogenicity of Env-based immunogens and an altered receptor usage *in vitro *[12,25,26,88]. Given the conservation of these sites in field strains, we predicted that one or both of these mutations may contribute to the process of cell culture adaptation which previous studies have shown to result in an increase in charge of the FIV Env V3-loop homologue and utilisation of CXCR4 alone as a viral receptor (CD134-independent infection) [27,33,34,89,90]. When the T271I and N342Y mutations were introduced into two primary field strains of FIV (GL8 and CPG41), the T271I mutation was shown to have a significant modulatory effect on the way the virus interacts with CD134. The GL8 and CPG41 strains of FIV recognise a complex determinant on CD134 that is dependent on residues in both CRD1 and CRD2 of the molecule, while strains such as PPR and B2542 are able to infect cells using a minimal determinant in CRD1 alone, suggesting a less complex interaction with CD134 [30,63,84]. That the removal of a single site for N-linked glycosylation (T271I) is sufficient to switch both GL8 and CPG41 from a complex to a minimal interaction would suggest that the N-linked glycosylation site ablated by the T271I mutation either forms a component of the receptor binding face of FIV Env, or contributes to the tertiary/quaternary structure of the Env trimer such that the receptor binding face of Env is presented to the viral receptor in the correct configuration. These data are reminiscent of studies with HIV-1 ADA where removal of an N-linked glycosylation site at the base of the V1V2 stem (N197) facilitated CD4-independent infection of canine cells through CCR5 [12], an effect that was postulated to be mediated by repositioning of the V1V2 loops and exposure of the CCR5 binding site [12]. An N197D mutation in Env improved CXCR4 utilisation by HIV ADA-1 following co-receptor switching from CCR5 to CXCR4 [91]. The mutations in V3 that conferred CXCR4 usage upon the virus were in themselves deleterious; it has been suggested that by altering the flexibility of the V1V2 loop, the N197D mutation compensated for the mutations in V3 that facilitated switching from CCR5 to CXCR4 usage [91]. Mutation of glycosylation sites adjacent to the V1V2 loops of HIV-1 DH12, a dual-tropic strain of HIV, either reduced or abolished completely cell-cell fusion mediated by both CXCR4 and CCR5 [92], this effect appeared to result, in part, from a marked reduction in CD4 binding by the mutated Env [92].

Several studies have demonstrated that the V1V2 region of HIV Env plays an important role in viral cell tropism and receptor binding [52-57]. For example, in early studies, mutations in the V1/V2 loop of HIV-1 HXBc2 were observed to reduce both CD4-binding capacity and syncytium formation [56] while subsequently, determinants in V1/V2 were found to contribute to CCR3 usage in that substitution of the V1/V2 loop of the dual-tropic (R3/R5) strain ADA into the R5 strain BaL rendered the Env dual-tropic [93]. Solution of the crystal structure of the deglycosylated gp120 core in complex with CD4 and antibody 17b indicated that the stem of the V1 and V2 loops of HIV Env contributes to the "bridging sheet", a four-stranded anti-parallel β-sheet that mediates chemokine receptor binding [7,94]. Binding of CD4 is thought to reposition the V1V2 loop stem (the V1V2 loop stem itself contacts CD4 [7]), facilitating the formation of the bridging sheet structure and subsequently permitting chemokine receptor binding. Thus modifications to the structure of the V1V2 loop may mimic CD4-binding and facilitate chemokine receptor binding. The T271I mutation in FIV Env may have a similar function; either by altering directly a contact point with CD134 or by facilitating CD134-independent infection. In this study, we found that the T271I mutation did not confer CD134-independent infection, rather it modulated usage of CD134 by the primary FIV Envs, reducing the requirement for residues in CRD2 of CD134 for viral entry and syncytium formation. A similar mode of interaction with CD134 has been described for the PPR and B2542 strains of virus, both of which are able to readily infect cells using CRD1 of CD134 alone [30,63]. Both the PPR [95] and B2542 [96] are capable of inducing immunological and neurological abnormalities *in vivo *that are consistent with immunodeficiency-causing lentiviruses, thus the ability of viruses to infect cells using CRD1 of CD134 alone is not in itself, debilitating to viral replication. It is possible that the nature of the interaction between the viral Env and CD134 changes with disease progression, in early infection viruses employ a high affinity complex interaction involving determinants in both CRD1 and CRD2 and in doing so may protect the virus from neutralising antibody or endogenous CD134-ligand (infection with PPR and B2542 is sensitive to modulation by CD134L [28]). As disease progresses, the Env-CD134 interaction may become less stringent, allowing the virus to expand more readily into CD134-negative cellular compartments through direct interactions with CXCR4. If N-linked glycosylation at N-269 contributes to the complexity of the Env-CD134 interaction *in vivo*, we would predict that late isolates of FIV would be more likely to harbour viral variants bearing mutations such as the T271I observed in FL4. Conversely, in early infection, viral variants bearing T271 would be suppressed. This hypothesis has been tested to some extent in a study in which cats were infected with the FL4 strain and virus re-isolated three years post infection [87]. Sequence analysis revealed an I271T reversion in 2 out of 3 cats and Y342N mutation in 3 of 3 cats [87], suggesting a strong selective pressure in favour of either reinstating the N-linked glycosylation sites following infection or suppressing the replication of T271I and Y342N bearing variants *in vivo*.

Neither the T271 nor the N342Y mutations affected the sensitivity of GL8 to neutralisation by sera from cats infected with the biological isolate of GL8, suggesting that this region may not be a primary target for neutralising antibodies *in vivo*. The N-linked glycosylation sites affected by the T271I and N342Y mutations in FIV lie in a predicted loop domain of FIV Env [76] that is analogous to the V1/V2 loop of HIV in schematic structural models [75]. V1 and V2 of FIV Env were originally assigned to hypervariable regions located in the leader and signal sequence regions of FIV Env [76], however, to facilitate ready comparisons between FIV and HIV we refer to the region between amino acids 248 and 354 as the V1/V2 homologue. The conservation of this region amongst viral strains is consistent with the region not being under a strong selective pressure from the host immune system. However as the Fel-O-Vax FIV vaccine is derived from FL4 virus bearing the T271I and N342Y mutations, it is possible that deglycosylation of this region in the vaccine strain may render the region immunogenic for vaccinates and contribute to protection from challenge.

In conclusion, we have shown that a single glycosylation site ablated in V1V2-loop homologue of FIV modulates the Env-CD134 interaction. As this glycosylation site is ablated in the FL4 vaccine strain, future studies should address whether antibodies induced by vaccination with immunogens bearing this mutation target this region of the Env and whether selective deglycosylation of this region may offer a strategy for the rationale design of FIV vaccines.

## Competing interests

The authors declare that they have no competing interests.

## Authors' contributions

EM and NL performed the DNA manipulations, cell culture, viral pseudotype preparation and biochemical analyses. AS carried out the virus neutralisation assays. BW purified mutant viruses and performed flow cytometric assays. MH and BW designed the experiments and wrote the manuscript. All authors read and approved the final manuscript.
